# Improving the structure and properties of whey protein emulsion gel using soluble interactions with xanthan and basil seed gum

**DOI:** 10.1002/fsn3.3598

**Published:** 2023-08-15

**Authors:** Mozhdeh Sarraf, Sara Naji‐Tabasi, Adel Beig‐Babaei, José E. Moros, M. Carmen Sánchez, José M. Franco, Adrián Tenorio‐Alfonso

**Affiliations:** ^1^ Department of Food Chemistry Research Institute of Food Science and Technology (RIFST) Mashhad Iran; ^2^ Department of Food Nanotechnology Research Institute of Food Science and Technology (RIFST) Mashhad Iran; ^3^ Department of Chemical Engineering, ETSI, Pro2TecS – Chemical Process and Product Technology Research Center Universidad de Huelva Huelva Spain

**Keywords:** emulsion gel, *Ocimum basilicum* L., soluble interaction, whey protein concentrate

## Abstract

Applying hydrocolloids in the structure of protein emulsion gel can improve its properties. Interaction of whey protein concentrate (WPC) (5%) with xanthan gum (XG) and basil seed gum (BSG) at different concentrations (0.2%, 0.4%, and 0.6%) was investigated to improve mechanical and structural properties of emulsion gel. Results illustrated that gums created a stronger structure around the oil droplets, which confocal images approved it. Also, the particle size decreased and uniformed by cooperating 0.6% gum in comparison with WPC (46.87 μm). The lowest and highest hardness values were observed in emulsion gel formed by WPC (1.27 N) and 0.6BSG: WPC (3.03 N), respectively. Also, the increase of gum concentration had a positive on consistency parameter of texture, so the value was 11.48 N s in WPC emulsion gel and it reached 0.6BSG: WPC (25.71 N s) and 0.6XG: WPC (19.96 N s). Evaluating the stability of the treatments by centrifugation indicated that 0.6BSG: WPC (89.10%) and 0.6XG: WPC (74%) had the highest level of stability. Increasing gum concentration increased the consistency and viscosity. Also, the viscoelastic properties of emulsion gel improved by 0.6% BSG. The elastic modulus of the WPC, 0.6XG: WPC, and 0.6BSG: WPC emulsion gels at the same frequency (1 Hz) was 240.90, 894.59, and 1185.61 Pa, respectively. In general, the interaction of WPC solution with hydrocolloids, especially BSG, is suggested to prepare more stable and elastic emulsion gels.

## Graphical abstract

The soluble complexes of whey protein and basil seed gum and xanthan gum improved the texture, particle size, zeta potential, microstructure, and viscoelastic properties of emulsion gel. The presence of gums resulted in the creation of a stronger structure, which could hold oil droplets well in the network. The presence of hydrocolloids created a stronger structure around the oil droplets, which confocal images approved it. The highest storage and loss modulus of emulsion gels were found for 0.6% basil seed gum.

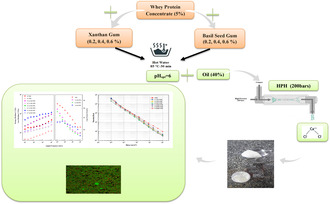

## 1 INTRODUCTION

Emulsions of oil‐in‐water are the basis of many industrial processes, although the emulsions are thermodynamically unstable systems (McClements, 2015). The soft‐solid materials called ‘emulsion gels’ comprise the aggregated emulsion droplets in a network (emulsion particulate gels). Many food products can be categorized as emulsion gels, including a wide range of food products such as cheese, ice cream, processed meat, spreads, desserts, and yogurt (Dickinson, 2012; Farjami & Madadlou, 2019). Emulsion gels, especially in the case of protein‐based oil‐in‐water emulsions, are generated with various methods; for instance, enzyme, high‐pressure treatment or homogenizations, salt or acid‐induced cold gelation (Dickinson, 2012).

Proteins can stabilize oil in water due to the presence of amino and carboxyl moieties that maintains surface activity (Hasenhuettl & Hartel, 2008; Isnaini et al., 2021). Whey proteins are widely used in the food industry due to their available, nutritional, and functional characteristics, such as emulsifier and gelling agent, thickening, and water‐binding capacity (Morr & Ha, 1993; Wang et al., 2023). By adsorbing whey proteins at the emulsion droplet surface, they create an electrostatic barrier against flocculation and coalescence and stabilize the system from approaching each other closely enough (Ye & Singh, 2006).

Although proteins show more tendencies to the oil phase due to their amphiphilic properties, in the absence of the aqueous phase, they cannot form strong bonds due to the limitation of their ability to expand with oil. On the other hand, polysaccharides only work well in aqueous solvents; they do not have enough molecular structure to form strong bonds with oil. Therefore, the simultaneous use of protein and polysaccharide will result in a cross‐linked structure at the oil–water interface (Patel, 2015; Romoscanu & Mezzenga, 2006; Wilde et al., 2004). The functional properties of protein–polysaccharide interaction depend on the molecular properties of each polymer and also on the nature of interactions between them (Sarraf et al., 2021). The combination of polysaccharides and proteins influences the system and generates thick and gel‐like absorbed layers charged (Brunchi et al., 2016; Sun et al., 2007).

One of the most favorite industrial hydrocolloids in the food industry is xanthan gum (XG) which is generated by *Xanthomonas campestris*. It is a heteropolysaccharide with branched chains, anionic characteristics, and a molecular weight of approximately 2 × 10^3^ kDa (Rosalam & England, 2006). High solubility in water due to the molecule polyelectrolyte nature of XG creates aqueous solutions with high viscosity, even at low concentrations. This gum is used as a gelling agent, a thickener stabilizer of emulsion in the food industry. Also, it causes the viscosity of the solution to decrease with increasing shear rate due to the shear‐thinning characteristics. XG solubility in cold water is intensified by the anionic side chains and also the gum imparts significant stability over wide pH, temperature, and salt content ranges as it is surveyed in different research (Murad et al., 2019). The great advantage of XG viscosity is stable even in the presence of large amounts of salts at high temperatures. It is due to the branching makeup of xanthan confers unusual rheological properties than other natural gums (Infee & Priyadharshini, 2015). Perez et al. (2010) reported that the interfacial functionality of WPC was improved by the synergistic interactions with XG, although WPC chemical complexity might complicate the elucidation of molecular events that govern adsorption dynamics of WPC/XG mixed systems at the air–water interface (Perez et al., 2010).

Basil seed gum (BSG) is a plant‐derived gum that has great potential to apply in the food industry. BSG, with scientific name *Ocimum basilicum*, improves high viscosity and shear‐thinning behavior because of its high molecular weight (2320 kg mol^−1^) (Hosseini‐Parvar et al., 2010; Naji‐Tabasi & Razavi, 2016). BSG can be applied in the food industry as a suspending agent, emulsion stabilizer, thickener, gelling agent, fat replacer, delivery system, and controlling crystal growth in the food industry (BahramParvar & Goff, 2013; Hosseini‐Parvar et al., 2015; Naji‐Tabasi et al., 2020; Naji‐Tabasi & Razavi, 2016; Zeynali et al., 2019). Basil is found worldwide and used in the food, pharmaceutical, and cosmetic industries (Calderón Bravo et al., 2021). The seeds have low calories and are a rich source of fiber and fatty acids. It also contains antioxidant compounds and many minerals, including vitamin and folic acid, β‐carotene, lutein, and zeaxanthin (Calderón Bravo et al., 2021; Gajendiran et al., 2016; Prakash et al., 2000). Behrouzain and Razavi (2020) reported that BSG can interact with whey protein below its pI and the lowest immiscibility was observed at pH of 6.0 and 7.0, 1:4 BSG: WPI, while at pH of 5.0, this happened for 1:9 BSG: WPI (Behrouzain & Razavi, 2020).

An important point in using hydrocolloids is their effect on the physicochemical and mechanical properties of the created system. Ghosh and Bandyopadhyay (2012) stated that proteins stabilize emulsions with the presence of polysaccharides due to the active surface through electrostatic interactions. Also, polysaccharides are responsible for controlling the rheological properties of the aqueous phase, such as gelation and changes in viscosity and emulsion stabilization (Ghosh & Bandyopadhyay, 2012). Behrouzain et al. (2020) reported that various biopolymer ratios of BSG: WPI can be applied for the creation of structure with a variety of different rheological and physicochemical characteristics (Behrouzain et al., 2020). Therefore, changing the concentration of polysaccharides has a significant effect on increasing the stability of emulsion gels.

Therefore, the aim of this study was to investigate the effect of soluble interaction of WPC–hydrocolloid (xanthan and basil seed gum) at different concentrations on the creation of more stable emulsion gel structure. Also, two different gums from different origins were compared. Different properties of emulsion gels including, microstructure, textural, rheological, droplet size, and stability were investigated.

## 2 MATERIALS AND METHODS

### 2.1 Materials

Basil seeds were purchased from a local market in Mashhad (IRAN). Whey protein concentrated (WPC) containing 70% (%wt) proteins (Westland, New Zealand) and xanthan gums (Sigma) were purchased. HCl, NaOH, and CaCl_2_ were supplied by Merck Co. Commercial sunflower oil (Iran) was used for the preparation of the emulsion gels. Deionizer water purified was used for the preparation of the solution. All the chemical materials were of analytical grade.

### 2.2 Preparation of BSG

The extraction was done according to Sarraf et al. (2021) study. First of all, the BS were cleaned, and mixed with distilled water in a ratio of 1:20. The seeds–water was heated in the water bath at 68°C for 20 min. The gum was separated from swelled seeds by an extractor by scrapping the mucilage layer on the seed surface. The BSG was precipitated by ethanol (three volumes of gum). The precipitated polysaccharides were dissolved in distilled water and then dried in an air‐forced oven at 40°C. The dried gum was milled and stored in a cool and dry place.

### 2.3 Preparation of emulsion gel by cold‐set method

For the preparation of soluble complexes, biopolymers were dispersed in deionized water. WPC solutions were prepared with a concentration of 5%. The WPC: gum solution was prepared at a constant concentration of WPC (5% w/w) and different concentrations of XG and BSG (0%, 0.2%, 0.4%, and 0.6% w/w). Sodium azide (0.02%) was added to prevent bacterial growth. The polymer solutions were stirred and then kept in the refrigerator for 24 h to completely hydrate. BSG:WPC and XG:WPC were mixed in a ratio of 1:1 and then the pH of the mixture was set to 6 by HCl and NaOH 0.1 N (pH meter Metrohm) (Sarraf et al., 2021). The solutions were placed in a heating circulator (Julabo, EH 19) at 85°C for 30 min and cooled immediately. According to Li and Xiang (2019), 40% sunflower oil was added to biopolymer solutions and prehomogenized with an ultraturrax (IKA, TG25) at 10,000 rpm for 2 min. After that, pressure homogenization (HL1.2, HST) was carried out by applying 20 MPa. After homogenization, the emulsion was placed in an ice bath to cool (Li & Xiang, 2019; Nayebzadeh et al., 2007). Finally, 10 mM CaCl_2_ was added to the emulsions and stirred. The emulsion gels were stored at 4°C for more investigations (Khalesi et al., 2019).

### 2.4 Particle size measurement

The droplet size of the emulsion gels was measured using a Master sizer (Malvern) after appropriate dilution at 25°C (Patel et al., 2014).

The mean diameter of the oil droplets as volume mean diameter (D[4,3]) and the droplet size distribution as span was calculated based on Equations 1 and 2, respectively.

(1) $$D=\left[4,3\right]\frac{\sum n_{i}d_{i}^{4}}{\sum n_{i}d_{i}^{3}}$$

where *n*_*i*_ is the number of particles of diameter *d*_*i*_.

(2) $$\text{Span}=\frac{d_{90}-d_{10}}{d_{50}}$$

where *d*_90_, *d*_10_, and *d*_50_ are the equivalent volume diameters at 90%, 10%, and 50% cumulative volume, respectively.

### 2.5 Microstructure studies

The optical microscopy technique (LX400, LABOMED) was utilized to study the microstructure of emulsion gels at ×20 magnifications. Emulsion gels were diluted with SDS sodium dodecyl sulfate 0.1%.

In addition, a confocal scanning laser microscope (CONFOCAL LEICA STELLARIS 8 STED, Leica Microsystems) was used to assess the emulsion gel microstructure. The emulsion gels were placed into a laboratory‐made welled slide and a coverslip (0.17 mm thickness). The coverslip is sealed with lacquer at its ends to prevent drying of the sample, ensuring that there was no trapped air gap or bubbles between the mixture and the coverslip. The mixtures were equilibrated for 5 min before testing. The glycerol immersion objective lens 93× was used to scan the emulsion gels (25°C) at ×20 magnifications. The CLSM was operated in fluorescence mode. Fluorescence from the sample was excited with a laser of 619 and 555 nm. The oil and the water phase were stained with (0.2 g/L in water) Fast Green and (0.1 g/L in water) Nile Red dye, respectively (Zembyla et al., 2018). Images were acquired and processed with Leica Application Suite X (LAS X) software. Each image contained 1024 × 1024 pixels.

### 2.6 Zeta‐Potential measurement

Zeta potential of emulsion gels was measured at 25°C by Malvern zeta sizer. The emulsion gels were diluted in deionized water. Zeta potential of particles was measured using a combination of the measurement techniques: electrophoresis and laser Doppler velocimetry sometimes called laser Doppler electrophoresis.

### 2.7 Characterization and stability of emulsion

#### 2.7.1 Centrifugation test

The stability of emulsion gels was evaluated with a centrifuge (Heidoph) for 10 min at 3500 rpm. For thermal stability, the samples were treated for 30 min at 80°C in the bath before centrifugation (Naji‐Tabasi et al., 2020) as Equation 3.

(3) $$\mathrm{ES}\left(\%\right)=\left(\frac{e_{v}}{t_{v}}\right)\times 100$$

Where *e*_*v*_ is the emulsion volume after centrifugation and *t*_*v*_ is the total volume of emulsion gel.

#### 2.7.2 Storage stability assay

The sample was stored in glass tube at 25°C for a month. The stability was determined every week (Naji‐Tabasi & Razavi, 2016).

### 2.8 Texture analysis

The textural properties of emulsion gels were studied using a texture analyzer (Stable Micro System, TA‐XT Plus) by back extrusion method. In this test, a flat (disc‐like) probe (England) with a diameter of 35 mm was used. The test was conducted with a depth of 10 mm and the rate of probe 1 mm s^−1^ with a trigger force of 3 g at the ambient temperature (Nourbehesht et al., 2018). The container used had a diameter of 45 mm. The parameters of hardness, consistency, adhesiveness, and apparent elastic modulus were evaluated.

### 2.9 Rheological measurements

Emulsion gels were characterized in a controlled‐stress RheoScope rheometer (Thermo Scientific) using a serrated plate and plate geometry (35 mm diameter and 1 mm gap).

Small amplitude oscillatory shear (SAOS) tests were carried out, inside the linear viscoelastic region, in a frequency range of 0.03–100 rad s^−1^ at 25°C. The results were investigated in terms of the storage modulus (G') and the loss modulus (G"). Stress sweep was conducted in a range of 0.01–1000 Pa (1 rad s^−1^, 25°C) to determine the linear viscoelastic range of emulsion gels.

### 2.10 Statistical analysis

The results were analyzed by a completely randomized factorial design according to ANOVA. Then, Duncan's multiple range test was applied to obtain the significant difference between means (*p* ≤ .05). The curves were plotted by Excel software 2013 and OriginLab software 2017.

## 3 RESULTS AND DISCUSSION

### 3.1 Particle size and morphological features

D[4,3], D[0.9], and the span of emulsion gels are summarized in Table 1. D[4,3] represents the volume average of the diameter of the particles. D[0.9] indicates that 90% of the drops of system are smaller than this diameter. Span is attributed to the distribution homogeneity of oil droplet size, and the smaller value indicates that the distribution of particles is more uniform (Drozłowska et al., 2020). The important point is the coalescence of oil droplets and it causes the span to increase in the emulsions (Campelo et al., 2017).

**TABLE 1:** Determination of zeta potential and particle size of emulsion gels stabilized with BSG:WPC and XG:WPC.^*^

Emulsion gel (%)	Parameters			
	Zeta potential (−mv)	D[4,3] (μm)	*d*_90_ (μm)	Span
WPC	22.40 ± 0.42^g^	17.80 ± 8.70^a^	71.08 ± 14.60^a^	46.87 ± 52.90 ^a^
0.2BSG:WPC	22.90 ± 0.38^f^	2.05 ± 1.15 ^g^	3.01 ± 1.99 ^g^	2.06 ± 2.10^g^
0.4BSG:WPC	26.30 ± 0.71^b^	11.50 ± 2.25^b^	43.9 ± 4.90 ^b^	4 ± 43.10^b^
0.6BSG:WPC	28.50 ± 0.80^a^	9.22 ± 0.52 ^c^	11.42 ± 0.98 ^e^	9.50 ± 1.10 ^d^
0.2XG:WPC	23.50 ± 0.81^e^	4.00 ± 1.15 ^f^	4.36 ± 0.50 ^f^	2.70 ± 0.80 ^f^
0.4XG:WPC	25.10 ± 0.28^d^	8.77 ± 1.20 ^d^	31.38 ± 1.20 ^c^	19.49 ± 2.50^c^
0.6XG:WPC	26.00 ± 0.64^c^	5.26 ± 0.57 ^e^	13.38 ± 2.01 ^d^	7.78 ± 0.60 ^e^

*Note*: Different letters indicate significant differences among emulsion gels in columns at *p* < .05 by Duncan's test.

* Max amount letter: a.

As shown in Table 1, D[4,3] ranged from 2 to 17 μm, where the highest value is obtained in WPC emulsion gel. The addition of BSG and XG to the emulsion gel helped to increase the viscosity of the continuous phase, decrease D[4,3], and inhibition of oil droplets flocculation. Rahmati et al. (2018) reported that emulsion without gums has the largest oil droplets at pH = 5; following that, repulsion between oil particles decreases, and coalescence has occurred. The size of protein molecules raise at pH close to IP (Isoelectric point) (Rahmati et al., 2018). The particle size is reduced because of increasing the repulsive force between gum and protein, which causes thermodynamic incompatibility between the two, and more polymer is placed on the surface of the oil droplets (Uruakpa & Arntfield, 2005). On the other hand, the higher viscosity of polysaccharides such as XG/BSG resulted in less movement and coalescence of emulsion droplets happened, and consequently, the droplet size decreased (Banaś & Harasym, 2021).

The raising gum contents from 0.2% to 0.4% resulted in an increment of oil droplets size due to the interaction between protein and polysaccharides so the results showed the D[4,3] of 0.2 and 0.4BSG: WPC were 2.05 and 11.5 μm, respectively. A similar result occurred in the XG: WPC emulsion gel and there was a significant difference between 0.2% (4 μm) and 0.4% (8.77 μm) XG. It indicates that there is a critical concentration that leads to the depletion flocculation in the continuous phase; following that, the emulsion gel will be unstable. It increases the adsorption force between the droplets due to the osmotic pressure between the particles, causing them to come closer to each other (Ghorbanian et al., 2015). Khorami et al. (2013) reported that the stability of emulsion decreased with increasing BSG from 0.15% to 0.3%, which 0.3% is the critical concentration in depletion flocculation and the reason for the emulsion instability (Khorami et al., 2013). The critical concentration of gum increased the particle size of the emulsion gel because of the excessive presence of unabsorbed anionic polysaccharides in the emulsion. On the other hand, higher concentration had a positive effect in reducing the speed and rate of collision of particles with each other and increased stability. The results were consistent with various observations (Khorami et al., 2013; Krstonošić et al., 2009; Laplante et al., 2005; Mohammadzadeh et al., 2013; Rahmati et al., 2018).

However, the particle size reached 9.22 and 5.26 in emulsion gel in the presence of higher concentration of BSG and XG (0.6%), respectively. Huang et al. (2001) investigated the particle size of emulsions prepared with different hydrocolloids. They reported that the particle size increased with increasing XG concentration from 0.05% to 0.5%. The increasing viscosity may reduce the efficiency of the homogenization process and leads to the formation of larger oil droplets (Huang et al., 2001).

By increasing the concentration of gums (0.6%), the particle size in BSG and XG gel emulsions reached 9.22 and 5.26 micrometers, respectively. The oil droplet size was lower than 4% gum concentration, but higher than 2% concentration. It is observed the particle size of WPC:XG emulsion was smaller than WPC: BSG, which may be related to the higher viscosity values of BSG compared to XG and weak homogenization efficiency. D[0.9] parameter of WPC emulsion gel was 71.08 μm, the highest among the samples (Table 1). This index decreased by adding 0.2% of gums. Creation soluble complex of WPC: 0.4% gums significantly increased D[0.9] of emulsions (*p* < .05). But D[0.9] decreased in emulsions containing WPC: 0.6% gum and reached 31.38 and 11.42 μm for XG and BSG, respectively.

WPC emulsion gel had the highest span (46.87), illustrating the particle size distribution in the sample without gum is not suitable. Adding gums at all concentrations had a positive influence on the creation of uniform particle size distribution.

The results of particle size indicate that the particle distribution in WPC: XG was more homogeneous than WPC: BSG. In general, emulsions with smaller particle sizes had more uniform oil droplets due to less flocculation of oil droplets in emulsions (Rahmati et al., 2018). Krstonošić et al. (2015) reported that XG is a nonabsorbent polymer with a high molecular mass. It reduces the average droplet diameter and improves the droplet size distribution as a result of increasing the viscosity of the aqueous phase and creating a network that reduces the droplet movement and the number of collisions (Krstonošić et al., 2015).

**FIGURE 1:**
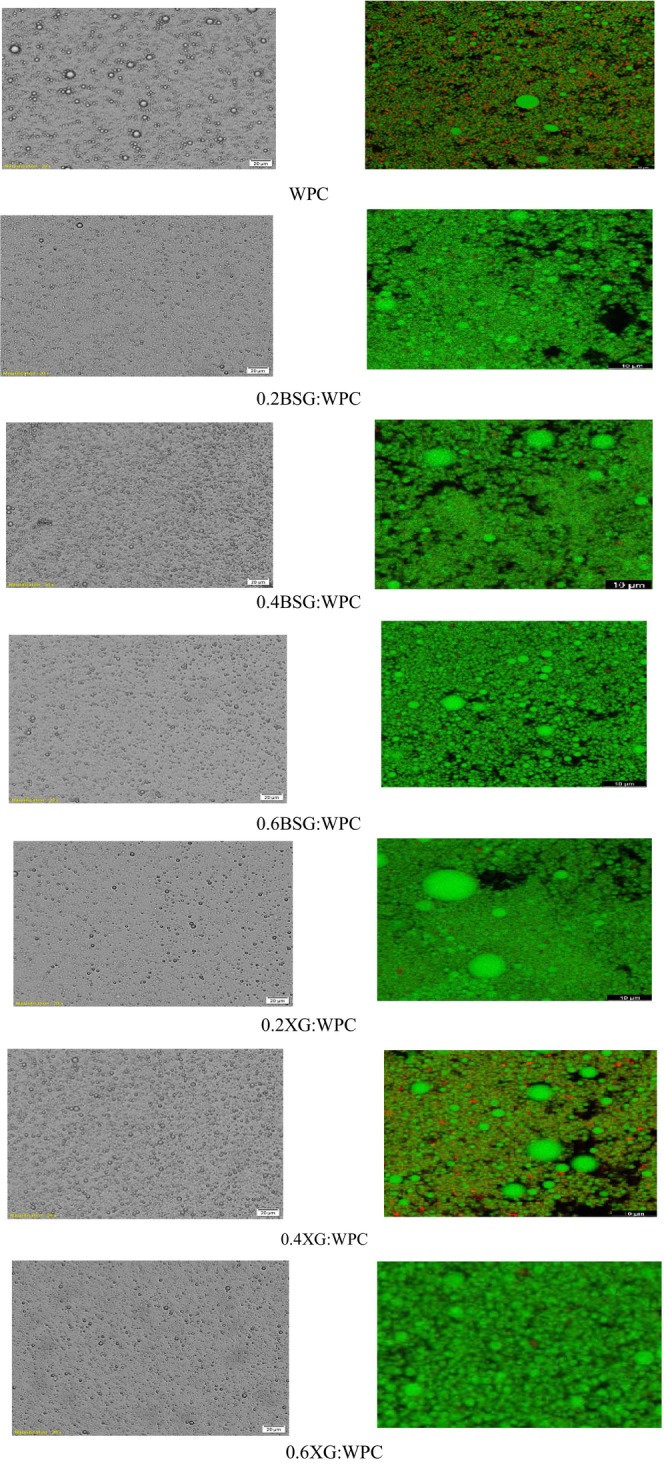
Optical (left) and confocal (right) microscopy images of emulsion gels.

Morphological features of emulsion gels (Figure 1) confirmed the result of particle size (Table 1). Biopolymer nature and composition, the emulsifying and stabilizing agent types and their concentration, the size of the droplets, oil concentration, and the viscosity of the water phase are important parameters affecting the emulsion gel microstructure (Mun et al., 2009). Figure 1 shows that the particle size of emulsion gels was similar to each other, except WPC. WPC emulsion gel showed larger droplet oils with nonuniform particle size distribution. The WPC emulsion had individual droplets with little flocculation. In addition, the oil droplets appeared slightly flocculated in the presence of gums; so, there were more empty spaces among droplets.

Confocal image (Figure 1, right) was another microscopy technique to investigate functions of hydrocolloids on microstructure emulsion gel, illustrating that Red Nile works well for the oil phase and Fast Green works well for biopolymer fraction. The black areas can be other compounds, bubbles, or uncolored spots that are unknown for regents. The microstructure of emulsion gel was shown as a function effect of hydrocolloids and the concentrations on the structure. In the emulsion WPC‐stabilized without gums, no sign of flocculation was observed and oil was not completely covered. The oil droplets were completely covered with an increasing amount of gum. At concentrations of 0.2%, oil droplets showed flocculation. Low xanthan gum concentration leads to depletion flocculation and increasing the xanthan concentration results in increasing the viscoelasticity of the aqueous phase (Nayebzadeh et al., 2007).

The results of BSG were similar to XG gum and the increase in particle size was observed at the concentration of 0.4%. On the other, there is not enough polysaccharide to cover all the droplets in the system at low concentrations of BSG, and large oil droplets are coagulated.

Osano et al. (2014) reported that an insufficient amount of protein and/or polysaccharide to coat oil droplets in an emulsion system after homogenization can lead to macromolecular bridging and increased particle size (Osano et al., 2014). Also, Banaś and Harasym (2021) confirmed that the higher the viscosity of polysaccharides such as xanthan gum/basil, the less the movement and coalescence of emulsion droplets and thus the smaller the droplet size (Banaś & Harasym, 2021).

### 3.2 Zeta potential

Table 1 shows the interaction between protein and polysaccharides caused a higher negative charge on the surface of polymer‐coated oil droplets. It means that more negative zeta potential values can be seen in all emulsion gel droplets as the polysaccharides increase. The absolute value of zeta potential increased from −22.40 to −28.60 mV by adding gum. This increase related to the interactions between WPC and BSG or XG, which was a sign of the formation of more negatively charged complexes with increasing polysaccharides concentrations (Zhao et al., 2015). The ζ‐potential of 0.6BSG: WPC and 0.6XG: WPC were −28.60 and −26.00 mV, respectively. The changes in charges were significant in both polysaccharides (*p* < .05). Although the trends of both hydrocolloids were similar, WPC: BSG had more negative charge compared to XG: WPC (Table 1). With the increase of polysaccharides concentration in soluble complex, the amount of zeta potential was driven toward a negative charge due to increasing the number of carboxyl groups on the polymer chains (Sarraf et al., 2021). The other researchers reported more negative zeta potential by increasing concentration of anionic gums in the emulsion gel (Hernández‐Marín et al., 2013; Rahmati et al., 2018). With the high zeta potential of emulsion gel (away from the point of the insoluble complex), the repulsive forces exceed the attractive forces, resulting in a relatively stable system (Lu & Gao, 2010).

Mirarab Razi et al. (2020) reported that BSG contains acidic functional groups such as uronic acid, which can cause a negative value of zeta potential in emulsion systems (Mirarab Razi et al., 2020). Also, there is glucuronic acid in the structural bone of XG, which can produce the same characteristics (Shang & Xiong, 2010).

### 3.3 Emulsion stability

Emulsion gel stability is used to describe the ability of an emulsion gel to resist alternations during storage (McClements, 2004). The effect of BSG and XG concentrations on the oil binding capacity of the emulsion gel samples was measured by the centrifuge method, and the results are shown in Table 2. Depending on the polymer concentration, time, and speed of centrifugation, three different structures can be created after centrifugation: (1) complete emulsion breakup with separated oil and water layers in the test tube; (2) partial emulsion breakup with a layer of separated oil at the top, a transparent water layer at the bottom and a layer of creamed emulsion in between; (3) no emulsion breakup with a creamed emulsion on top of the transparent water layer (Krebs et al., 2012). In all emulsion gels, release of free water with no oil happened. The amount of free water declined with increasing polysaccharide concentrations according to the results obtained from hardness of the structures. It represents WPC alone could not form a stable emulsion gel, and polysaccharides had a positive effect on complex stability.

**TABLE 2:** Determination of storage, centrifuge, and thermal stability of emulsion gels stabilized with BSG:WPC and XG:WPC.^*^

Emulsion gel (%)	Parameters					
	Loss of oil during storage (%)				Centrifuge stability (%)	Thermal stability (%)
	Week 1	Week 2	Week 3	Week 4		
WPC	4	11	20	24	31.36 ± 0.03^g^	26.60 ± 0.10^f^
0.2BSG:WPC	–	–	>1	~1	69.10 ± 0.10^d^	68.40 ± 0.20^c^
0.4BSG:WPC	–	–	>1	~1	79.98 ± 0.05^b^	68.82 ± 0.09^c^
0.6BSG:WPC	–	–	–	~1	89.10 ± 0.10^a^	74.00 ± 0.50^a^
0.2XG:WPC	–	>1	>1	1	49.00 ± 0.05^f^	47.95 ± 0.70^e^
0.4XG:WPC	–	‐	>1	>1	62.18 ± 0.01^e^	56.38 ± 0.50^d^
0.6XG:WPC	–	–	–	>1	74.00 ± 0.10^c^	71.30 ± 0.40^b^

*Note*: Different letters indicate significant differences among emulsion gels at *p* < .05 by Duncan's test.

* Max amount letter: a.

The results of the emulsion gel stability in BSG: WPC and XG: WPC determined that hydrocolloids increased the stability of emulsion gels. WPC emulsion gel had 31.36% stability after centrifugation which could be related to the particle size. D[4,3] of WPC emulsion gel was 17.8 μm, and the high size of droplets caused hardness and consistency reduction. It is reported that there is the critical gum concentration that leads to the formation an unstable structure and the separation of the serum phase (Khorami et al., 2013). The result of centrifuge stability illustrated that WPC: BSG had more ability to prevent the creaming of emulsion gel in comparison with WPC: XG. The most stability was observed in 0.6BSG: WPC (89.10%).

The surface activity of proteins and the presence of polysaccharides help to stabilize emulsion gels through electrostatic interactions. Polysaccharides are also responsible for controlling the rheological properties of the aqueous phase, such as gelation and change in viscosity and stabilization of the emulsion (Ghosh & Bandyopadhyay, 2012). Therefore, changing the concentration of polysaccharides has a significant effect on increasing the stability of emulsion gels.

In the thermal stability method, the same trend was observed in all hydrocolloid concentrations. The heated emulsion gels released more free water. The emulsion gel containing WPC had the least stability against stress like the centrifuge, while with increasing hydrocolloids, the resistance of emulsion gels significantly increased. The strongest system was related to the emulsion gels with the highest hydrocolloid percentages due to creating stronger network and better trapping oil droplets. The stability of BSG in all concentrations was higher than XG (Table 2).

Although the centrifuge assay is the way to investigate emulsion gel stability rapidly, the reaction of emulsion gel during storage is important. Creaming is one of the instabilities of oil‐in‐water emulsions caused by the density difference between the two liquids in the emulsion and the gravitational force (Tadros, 2004). Creaming usually occurs in emulsions that contain a nonabsorbing polymer due to a deficient flocculation mechanism (McClements, 2000). Several studies have reported that when the polysaccharide concentration is above a certain concentration, the droplets tend to flocculate, which leads to the formation of a three‐dimensional gel‐like network of emulsion droplets and delays creaming beyond a critical concentration (Krstonošić et al., 2009; Naji‐Tabasi et al., 2021). The evaluation of structure stability was done during storage at ambient temperature and the evidence showed that a decrease in the height of the gel emulsion was observed in all samples. As shown in Table 2, the emulsion gel of WPC had the lowest power to maintain structure in the first week which can be related to larger particle size of WPC emulsion gel, its weak network gel, and lower viscosity of the continuous medium. The amount of oil loss of WPC emulsion gel from the first to fourth week reached 4%, 11%, 20%, and 24%, respectively. On the other hand, WPC: 0.6BSG and WPC: 0.6XG were stable until 21 days and during the last week ~1% oil was observed on the surface of the emulsion gels. Khorami et al. (2013) investigated the stability of BSG emulsions during storage. They reported that the droplet size gradually increased after 20 days (Khorami et al., 2013). This phenomenon caused the formation of clots and the creation of two phases in the structure of emulsions.

Emulsion gels containing 0.2% and 0.4% XG showed sufficient stability in the first week. After 2 weeks, WPC: 0.2XG lost its stability, and oil droplets appeared on the surface of the gel emulsion. The WPC: 0.4XG sample was stable in the third week.

Increasing the polysaccharide concentration resulted in the formation of a stronger network than the samples with lower concentrations. The important point is that BSG was more stable against stress and heat, while XG showed relatively better stability during storage. However, the difference during storage was negligible.

In general, the aqueous phase plays a pivotal role in stability of emulsion gels against creaming. Niu et al. (2016) reported a decrease in droplet movement with increasing BSG concentration because of an increment of emulsion viscosity (Niu et al., 2016). Also, it can improve emulsion stability by reducing the surface tension at the oil–water interface due to its surface active properties (Mirarab Razi et al., 2020). The emulsion droplets tend to merge with adjacent droplets when they collide with each other and lead to change of droplet size during storage (McClements, 2004). Most hydrocolloids prevent droplet coalescence through viscosity modification or gelation of the continuous phase (Dickinson, 2003; Naji et al., 2012). It was expected that higher viscosity of BSG and the presence of WPC create a more suitable protective layer on the droplet surface compared to XG during storage. However, as mentioned, the difference between these two gums was not significant in terms of stabilization over time. But, the resistance of WPC: BSG emulsion gel against centrifugation and thermal treatment was more considerable than WPC: XG.

### 3.4 Textural properties

Table 3 shows the impact of different concentrations of BSG and XG on the textural properties of WPC emulsion gels.

**TABLE 3:** Determination of textural properties of emulsion gels stabilized with BSG:WPC and XG:WPC.^*^

Emulsion gel (%)	Hardness (N)	Adhesiveness (N s)	Consistency (N s)	Apparent elasticity modulus (N s^−1^)
WPC	1.27 ± 0.21^g^	−5.79 ± 0.24^a^	11.47 ± 0.35^g^	0.20 ± 0.03^d^
0.2 BSG:WPC	2.19 ± 0.21^d^	−8.66 ± 0.20^c^	18.20 ± 0.18^d^	0.40 ± 0.01^b^
0.4 BSG:WPC	2.51 ± 0.18^c^	−11.25 ± 0.12^d^	20.52 ± 0.21^b^	0.77 ± 0.04^a^
0.6 BSG:WPC	3.03 ± 0.19^a^	−15.14 ± 0.27^g^	25.71 ± 0.20^a^	0.76 ± 0.05^a^
0.2 XG:WPC	1.71 ± 0.12^f^	−7.76 ± 0.38^b^	15.06 ± 0.20^f^	0.21 ± 0.03^d^
0.4 XG:WPC	2.18 ± 0.27 ^e^	−12.61 ± 0.40^e^	17.95 ± 0.47^e^	0.21 ± 0.01^d^
0.6 XG:WPC	2.83 ± 0.32^b^	−13.10 ± 0.18^f^	19.96 ± 0.30^c^	0.32 ± 0.01^c^

*Note*: Different letters indicate significant differences among emulsion gels at *p* < .05 by Duncan's test.

* Max amount letter: a.

WPC emulsion gel had the lowest harness, which is related to its weak gel network. The hardness and consistency of emulsion gels increased with increment of hydrocolloid concentration (*p* > .05). The emulsion gels containing 0.6 BSG obtained the most hardness (3.03 N) and consistency (25.71 N s). It was found that samples prepared with the higher BSG concentration resulted in better network structure and more trapping oil droplets compared to samples of lower concentrations (Meng et al., 2018). The trend in emulsion gels containing different concentrations of XG was similar to BSG and the parameters like hardness and consistency increased with increasing XG concentration. The results were consistent with the examination of the droplet size distribution and the compression of the emulsion gel structure, so the size of the emulsion gel droplets was estimated to be smaller as the polysaccharide concentration increased. Published research shows that the hardness of texture depends on the concentration of biopolymers (Nishinari et al., 2013; Schober et al., 2005). On the other hand, Farahmandfar et al. (2017) investigated the textural properties of emulsions with the same concentration of three hydrocolloids (cress seed gum, quince seed gum, and BSG) and the reports indicated that BSG had the highest hardness among treatments (Farahmandfar et al., 2017).

WPC and WPC: 0.6BSG emulsion gels had the lowest (−5.79 N s) and highest (−15.14 N s) adhesiveness, respectively. Overall, the concentrations of BSG had more hardness and adhesiveness compared to XG. However, the trend of both hydrocolloids was similar (Table 3) and the differences were significant (*p* < .05). On the other hand, appearance modulus of elasticity increased with increment of gum concentration.

As shown in Table 3, although hardness increased with increasing hydrocolloid concentration, adhesiveness also increased. Nishinari et al. (2013) reported that a high percentage of XG increased the viscosity of aqueous xanthan solutions. Therefore, the WPC sample without hydrocolloid had the lowest adhesiveness (−5.79 N s), while this parameter is −15.14 and −13.10 N s in the sample containing 0.6% of BSG and XG, respectively.

In general, at the same concentration of hydrocolloids, BSG had more hardness and adhesiveness compared to XG, and the differences were significant (*p* < .05), although the trends of both hydrocolloids were similar to each other.

The apparent modulus of elasticity, which is an indicator of the stiffness in the texture from emulsion gel, showed an increasing trend with increasing gum concentration. The amount of the G' and G" modulus was also higher in BSG compared to XG. The value of apparent modulus of elasticity in the emulsion gel containing 0.6XG: WPC was 0.32 N s^−1^, whereas 0.2%–0.6% BSG: WPC had a much higher value (0.40–0.76 N s^−1^).

### 3.5 Rheological properties

**FIGURE 2:**
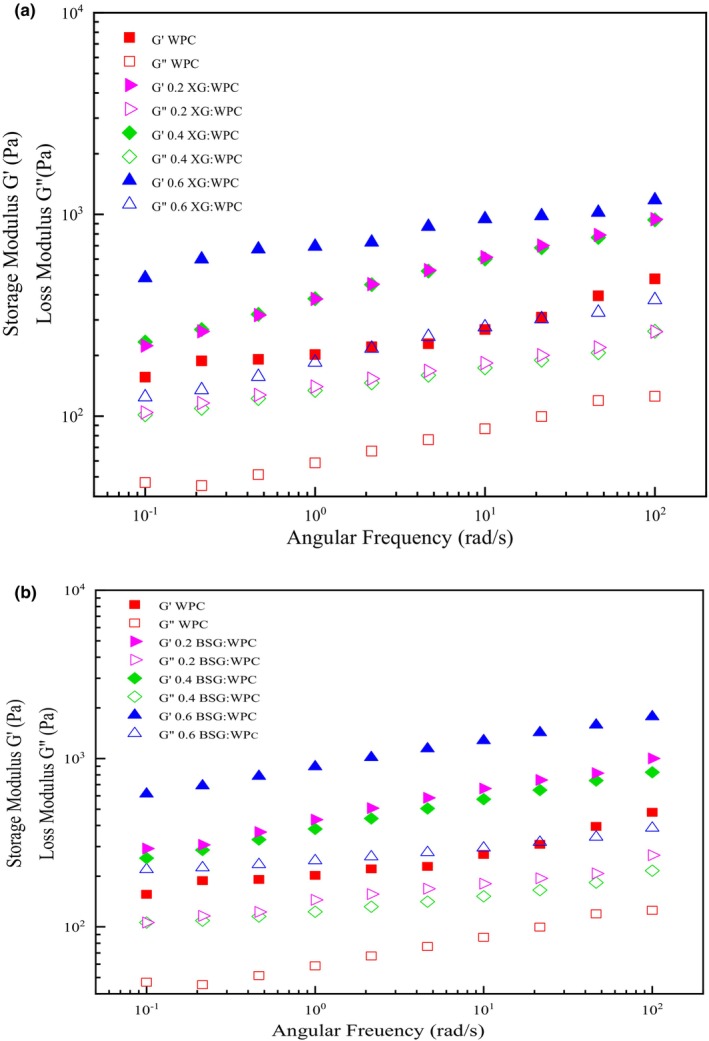
The frequency sweep curves of emulsion gels stabilized with XG:WPC (a) and BSG:WPC (b).

There are three types of systems in frequency sweep evaluation: if G' > G" is, it indicates that the sample is gel, G" > G' is related to dilute solutions they are close to each other at higher frequencies, and for concentrated systems, G' < G" at low frequency and crossover each other in middle of the frequency range (Shahbazizadeh et al., 2022). According to Figure 2, the storage modulus (G') was higher than the loss modulus (G") in all samples, indicating the elastic property dominated the viscous behavior of the emulsion gels. This behavior could be induced during time by Ca^+2^ at refrigerator temperature and a gel formed (Khalesi et al., 2019). The storage and loss modulus were slightly close to each other at high frequencies, but no crossover was observed, which shows that G' and G" have a weak frequency dependence.

The storage and loss moduli, the complex viscosity, and the loss tangent of the different emulsion gels obtained at 1 Hz are compared in Table 4. The viscoelastic properties of emulsion gel improved by using WPC‐gums. G' and G" increased by the increment of XG concentration. Nayebzadeh et al. (2007) reported that elastic modulus increased at low xanthan concentration due to segregative phase separation and strengthen protein structure. On the other hand, a much higher xanthan concentration leads to a lack of protein clusters and declining gel strength. Increasing the concentration of BSG leads to an increase in molecular interaction and the promotion of self‐aggregation phenomena of molecular chains. As a result, the higher concentration of BSG in the emulsion improves the viscoelastic properties (Hosseini‐Parvar, 2009). G' and G" of WPC: 0.4% gums were less than 0.2% and 0.6%. Sarabi‐Aghdam et al. (2021) investigated the effect of BSG concentrations on whey protein‐isolated gels. They reported that increment of BSG concentration from 0 to 0.3% (w/v), G' and G" significantly increased and it indicates that strength of structures is significantly improved (Sarabi‐Aghdam et al., 2021).

**TABLE 4:** Dynamic rheological parameters of emulsion gels determined by frequency sweep test at *f* = 1 Hz and 25°C.^*^

Emulsion gel (%)	G'(Pa)	G″(Pa)	*η* ^*^(Pa s)	Tanδ
WPC	240.9 ± 3.70^g^	79.29 ± 0.90^f^	40.38 ± 2.10^f^	0.33 ± 0.04^a^
0.2 XG:WPC	554.35 ± 0.90^d^	172.37 ± 1.10^c^	92.44 ± 1.00^d^	0.31 ± 0.04^b^
0.4 XG:WPC	547.18 ± 1.60^e^	163.75 ± 0.60^d^	90.95 ± 1.30^d^	0.29 ± 0.02^c^
0.6 XG:WPC	894.59 ± 2.40^b^	256.7 ± 2.00^b^	148.20 ± 2.40^b^	0.24 ± 0.01^e^
0.2 BSG:WPC	608.43 ± 2.20^c^	171.97 ± 0.80^c^	100.94 ± 0.95^c^	0.28 ± 0.02^cd^
0.4 BSG:WPC	525.6 ± 2.50^f^	144.13 ± 2.00^e^	86.78 ± 0.99^e^	0.27 ± 0.02^d^
0.6 BSG:WPC	1185.61 ± 9.20^a^	282.19 ± 3.30^a^	194.06 ± 3.60^a^	0.24 ± 0.02^e^

*Note*: Different letters indicate significant differences among emulsion gels at *p* < .05 by Duncan's test.

* Max amount letter: a.

Also, emulsion gels containing BSG had higher G' and G" than XG‐based emulsion gels at the same concentration. The droplet size of WPC: 0.6BSG emulsion gel was the smallest. Polysaccharides can cover more surface area of the droplets, by the reduction of oil droplets size and increasing the polysaccharide concentration (Xu et al., 2020). The increasing coverage of droplets prevents the approaching of oil droplets and a stronger three‐dimensional network is created. Paul (1996) reported that droplet size has a significant effect on the rheology of emulsions. Emulsions have a much higher viscosity and storage modulus than coarse emulsions if they have small droplet size (due to the reduced distance between oil droplets and a greater tendency to clot) and the pseudoplastic effect is much stronger in fine emulsions (Paul, 1996). The lowest values of G' and G" were observed for the WPC (gum‐free) emulsion gel, due to its high particle size.

Tanδ shows a ratio of loss energy to storage energy in each cycle. If tanδ is more than 1, elastic behavior is prominent in the emulsion gel, and if it is less than 1, it indicates that viscous behavior of the emulsion gel is prominent. The tanδ value higher than 0.1 is also indicating not being a real gel, it means that the structure is between high concentrated biopolymer and a real gel (Mandala et al., 2004). The range of tanδ values of the emulsion gels was 0.24–0.33 indicating that all emulsions did not have a real gel structure, applying a high shear rate could easily disrupt them. This confirms the behavior of the weakened flow by cutting the samples. Therefore, these emulsion gels can be described as weak gels (Mandala & Sotirakoglou, 2005). The 0.6BSG: WPC obtained the highest value of tanδ which is associated with the formation of a stronger intertwined network in these samples (Jaberi et al., 2020). The most angle value was related to WPC (0.33). The entanglement of chains and macromolecule links is unstable due to unrealistic gel. The viscoelastic properties of emulsion gels are dependent on their particle size, so it was reported in researches that emulsions with small particle sizes have higher yield stress, viscosity, and storage modulus than emulsions with large particle size (Naji‐Tabasi & Razavi, 2016; Otsubo & Prud'homme, 1994; Pal, 1996).

The complex viscosity (η*) value of the emulsion gels containing 0.6% gum was higher in comparison with the emulsion gels that had lower contain gums. η* of WPC was 40.38 Pa s, while the complex viscosity of WPC: BSG and WPC: XG was 194.06 and 148.20 Pa s, respectively.

Table 5, G' and G" frequency dependency was investigated according to the power‐law exponents *b* (elastic properties of the gels) and *d* (viscosity properties of the gels) in *α*_1_ *ω* ^*b*^ and *α*_2_ω^d^, respectively (Table 5). At *b* and *d* values close to zero, gels had a slight dependence on frequency. According to the results obtained, *b* and *d* exhibited slight frequency dependency to frequency, which is characteristic of physical gels (Khondkar et al., 2007; Yoneya et al., 2003).

**TABLE 5:** Power‐law parameters calculated for the storage and loss moduli of emulsion gels (frequency sweep test, *τ* = .1 Pa, 25°C).^*^

Emulsion gel (%)	*G*' *= α*_1_ *ω* ^*b*^			*G*″ *= α*_2_ *ω* ^*d*^		
	*α*_1_	*b*	*R* ^2^	*α*_2_	*d*	*R* ^2^
WPC	698.07 ± 14.7^b^	0.11 ± 0.007^e^	.962	180.07 ± 14.7^ab^	0.15 ± 0.007^a^	.990
0.2 XG:WPC	377.44 ± 5.5^e^	0.20 ± 0.004^a^	.996	144.59 ± 3.16^bc^	0.12 ± 0.007^bc^	.971
0.4 XG:WPC	377.34 ± 5.69^e^	0.19 ± 0.004^ab^	.996	131.61 ± 3.7^c^	0.13 ± 0.01^b^	.967
0.6 XG:WPC	698.07 ± 14.71^b^	0.11 ± 0.007^e^	.972	185.22 ± 3.56^ab^	0.16 ± 0.006^a^	.990
0.2 BSG:WPC	431.17 ± 2.1^c^	0.18 ± 0.006^bc^	.960	138.34 ± 3.98^a^	0.13 ± 0.009^b^	.960
0.4 BSG:WPC	382.58 ± 2.7^d^	0.17 ± 0.002^c^	.998	124.01 ± 2.7^c^	0.11 ± 0.007^c^	.964
0.6 BSG:WPC	892.40 ± 5.78^a^	0.15 ± 0.002^d^	.998	250.96 ± 3.26^a^	0.08 ± 0.005^d^	.970

*Note*: Different letters indicate significant differences among emulsion gels at *p* < .05 by Duncan's test.

* Max amount letter: a.

## 4 CONCLUSIONS

The results showed that the creation of the soluble complex of BSG and XG with WPC improves the emulsion gel stability. The polymer covered oil droplets and prevented their coalescence. Increment of BSG and XG concentration increased the hardness and elastic properties of emulsion gels. In terms of rheology properties, all emulsion gels had shear thinning and weak gel behavior. 0.6BSG: WPC with higher viscosity and storage and loss moduli exhibited the best emulsion structure (size distribution) and stability. BSG had more positive effects than XG, which may be related to more flexibility, more protein content, and higher viscosity of BSG. There are other features that make the use of basil gum more preferable, such as its more accessibility, natural origin, and easy extraction.

## AUTHOR CONTRIBUTIONS

**Mozhdeh Sarraf:** Formal analysis (equal); investigation (equal); methodology (equal); writing – original draft (equal). **Sara Naji‐Tabasi:** Conceptualization (equal); methodology (equal); project administration (equal); supervision (equal); validation (equal); writing – review and editing (equal). **Adel Beig‐Babaei:** Project administration (equal); supervision (equal); validation (equal); writing – review and editing (equal). **José E. Moros:** Project administration (equal); supervision (equal); writing – review and editing (equal). **M. Carmen Sánchez:** Project administration (equal); validation (equal); writing – review and editing (equal). **José M. Franco:** Project administration (equal); writing – review and editing (equal). **Adrián Tenorio‐Alfonso:** Writing – review and editing (equal).

## FUNDING INFORMATION

No funding.

## CONFLICT OF INTEREST STATEMENT

The authors declare they have no competing interests.

## CONSENT FOR PUBLICATION

All authors have read and agreed to the published version of the manuscript. All authors read and approved the final manuscript.

## Data Availability

The authors confirm that the data supporting the findings of this study are available within the article.
